# Lincoln as a Peacemaker

**Published:** 1882-11

**Authors:** 


### LINCOLN AS A PEACE-MAKER.

[From the Philadelphia Ledger.]

The Rev. Dr. W. N. Miner, of Trenton, N. J., relates an anecdote of Abraham Lincoln which places him among the peace-makers. Dr. Miner was a resident in Springfield, and gives the narrative in the words of another resident: "I had some trouble with a neighbor, and I determined to go to law with him. When I got to Lincoln's office I found my opponent was already there and had stated his case. I told my side of the story, and Lincoln then said: 'You have a good case to go to court; but if you do you will both lose all your money and make yourselves life-long enemies. Why don't you settle the matter right here?' We argued for some time without coming to an agreement, and finally Lincoln said: 'Well, I'm going to dinner. Try and settle it before I get back; and, in order that you may not be disturbed, I'll lock the door.' He locked us in and left us there, but when he returned we had compromised our trouble."

